# Exploring the Role of Telemedicine in Duchenne Muscular Dystrophy: Benefits and Challenges

**DOI:** 10.2196/77698

**Published:** 2025-09-19

**Authors:** Eliza Wasilewska, Andrzej Wasilewski, Alessandro Onofri, Jan Wasilewski, Dominika Sabiniewicz-Ziajka, Agnieszka Sobierajska-Rek, Jarosław Meyer-Szary, Karolina Śledzińska, Jolanta Wierzba, Marek Niedoszytko, Sylwia Małgorzewicz

**Affiliations:** 1 Department of Allergology and Pulmonology University Clinical Center Gdańsk (Accredited Duchenne Center) Medical University of Gdańsk Gdansk Poland; 2 Student Scientific Association of Medical Chemistry and Immunochemistry Wroclaw Medical University Wrocław Poland; 3 Pediatric Pulmonology & Cystic Fibrosis Unit Respiratory Intermediate Care Unit, Sleep and Long-Term Ventilation Unit Bambino Gesù Children’s Hospital, Scientific Institute for Research Rome Italy; 4 Rochester Institute of Technology Rochester, NY United States; 5 Department of Rehabilitation Medical University of Gdańsk Gdańsk Poland; 6 Department of Pediatric Cardiology University Clinical Center (Accredited Duchenne Center) Medical University of Gdańsk Gdańsk Poland; 7 Department of Paediatrics, Haematology and Oncology University Clinical Center (Accredited Duchenne Center) Medical University of Gdansk Gdańsk Poland; 8 Department of Clinical Nutrition University Clinical Center Gdańsk (Accredited Duchenne Center) Medical University of Gdańsk Gdańsk Poland

**Keywords:** neuromuscular disorders, Duchenne muscular dystrophy, telemedicine, eHealth, digital medicine

## Abstract

Duchenne muscular dystrophy (DMD) is the most frequent, progressive disease caused by a genetic defect that leads to the production of a nonfunctional form of dystrophin, thereby causing premature death. Ways to improve, adapt, and facilitate the care of people with DMD are still being explored. This viewpoint, developed by an accredited Duchenne center, aims to present current telemedicine options specifically tailored for patients with DMD and to discuss the advantages and limitations of these approaches across various health care domains. As one of the first centers in Poland to implement such an approach, the accredited Duchenne center provides targeted home-based care by using digital platforms and telemedicine tools. Additionally, we explore the potential of telemedicine to support different types of remote communication, including provider-to-provider, between patient/caregiver and provider, and between patient/caregiver and patient/caregiver interactions. This model has the potential to significantly enhance access to specialized care and improve the continuity and quality of life for those living with DMD.

## Introduction

A patient with Duchenne muscular dystrophy (DMD) has unique specific needs since the disease is not only chronic but also progressive and leads to premature death in the second or third decade of life. The essence of DMD is progressive muscle weakness caused by a genetic defect that leads to the production of a nonfunctional form of dystrophin. As a result, muscle cells are irreversibly damaged [1,2].

To date, there is no effective causal treatment for patients with DMD. Gene therapies such as exon skipping, stop codon read-through, or adeno-associated virus gene therapy are promising but limited by many factors, for example, the type of mutation [3]. Therefore, the basic treatment still remains—apart from corticosteroid therapy, multidisciplinary specialist care. Due to the interplay of muscles and organs, an interdisciplinary care team (according to the guidelines) should include a neurologist, pulmonologist, physiotherapist, cardiologist, dietitian, psychologist, endocrinologist, and other health workers [4,5]. The frequency of the follow-up visits depends on the patient’s clinical condition (at least once a year), but the increase in the survival rate has resulted in an increase in the severity of the disease and the need for more frequent contact with health care professionals [1,2,4]. This may be inconvenient or impossible, particularly for people with reduced mobility and/or who live a long way from the research center. Ways to improve, adapt, and facilitate the care of people with DMD are still being explored.

One way may be the use of eHealth, which is a rapidly growing field and can support many health care processes. As of 2012, only 31% of the hospitals and 15% of the outpatient clinics were using telemedicine services in the European Union [6,7]. Currently, digital health interventions are available for most people in the United States, Europe, Canada, and Australia [8,9]. Notably, the pandemic was a catalyst for the development of new forms of virtual medical care, and it significantly accelerated the introduction of telemedicine in patients with DMD.

This up-to-date viewpoint based on the experience of our accredited center and collaboration with other centers for children with DMD aims to provide current telemedicine options dedicated to patients with DMD and discusses the pros and cons in different health care areas. We also explore the possible use of telemedicine services at a distance for communication conducted between 2 or more providers (provider-to-provider telemedicine), between patients/caregivers and providers (client-to-provider telemedicine), and between 2 or more patients/caregivers (client-to-client telemedicine).

In this paper, we have focused only on telemedicine due to its rapid development and increasing widespread use, even though it is only one of the components of the broadly understood eHealth according to the World Health Organization (WHO) [10].

## Types of Telemedicine

### Client-To-Provider Telemedicine

The rapid increase in the use of telemedicine during the COVID-19 pandemic was evident, and many countries implemented individualized DMD telecare approaches [11-16]. For example, in Italy, remote clinical evaluation and administration of functional scales were lacking for neuromuscular diseases before the pandemic [17], but these increased rapidly during the pandemic. Similarly, in France, teleconsultation guidelines underwent rapid changes, allowing various forms of teleconsultation, including telephone, whereas videoconferencing was the primary method used before the pandemic [18]. In Norway, at the same time, 14% of the medical professionals used telemedicine to contact a patient with a rare disease and monitor its development during the pandemic [19].

Many well-organized telemedicine programs have been related to other neuromuscular diseases. In 2014, in Massachusetts, TelePALS (Telemedicine for People with Amyotrophic Lateral Sclerosis) was performed with follow-up structured care via video televisits [20]. In another study in 2019, a trained nurse traveled to patients’ homes and performed and recorded video assessments for later review by members of the multidisciplinary team [21]. Yet another project in 2018 and 2020 included a mobile app for weekly surveys about symptoms, complications, noninvasive ventilation use, and gastrostomy use to communicate with clinic staff [22,23]. These projects showed that telemedicine is feasible and acceptable to both health care professionals and patients with amyotrophic lateral sclerosis. A telemedicine program in 2018 for another neuromuscular disease, facio-scapulo-humeral muscular dystrophy, has also shown improvement in the quality of life and the possibility of reducing hospitalizations [24].

### Provider-To-Provider Telemedicine

Telemedicine has opened up opportunities for remote connections between health care professionals. The e-community of health care providers has become a common tool not only for scientific purposes but also for everyday clinical practice involving patients with DMD [25]. In Italy, during the COVID-19 pandemic, efforts were made to create a network for professionals and less-experienced doctors to enhance their knowledge of DMD [26]. This network also facilitated education through webinars and collaboration among distant researchers, projects, and clinical trials. Collaborative efforts between different centers to create large databases have helped in developing new medicines and policies for rare diseases. As of May 14, 2023, over 300 clinical trials on DMD have been registered on the clinical trial platforms (clinicaltrials.gov and clinicaltrialsregister.eu).

**Figure 1:**
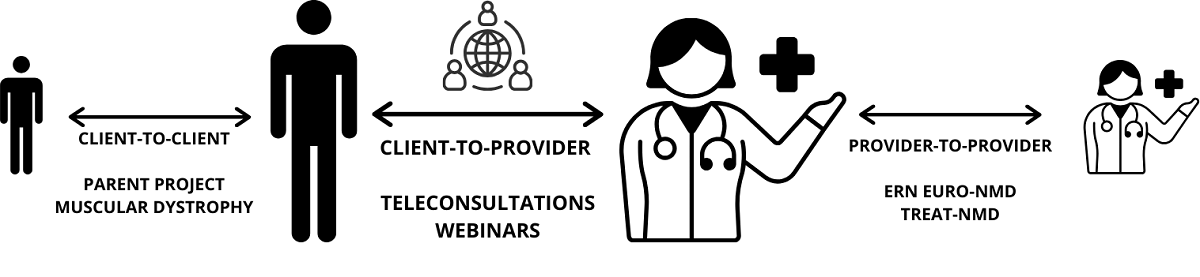
EURO-NMD (European Reference Network for the thematic grouping of rare Neuromuscular Diseases). ERN: European Reference Network; NMD: neuromuscular disease.

Another example of collaborative cooperation is the European Reference Networks established in 2017. These networks connect medical specialists across different disciplines through a dedicated information technology platform and telemedicine tools, effectively forming a virtual advisory board. EURO-NMD (European Reference Network for the thematic grouping of rare neuromuscular diseases) is a European Reference Network dedicated to rare neuromuscular diseases, aiming to facilitate diagnostic processes, standardize therapeutic approaches, and improve the efficiency and accessibility of health care systems [27] (Figure 1).

### Client-To-Client Telemedicine

Nearly 2 decades ago, the first teleproject was proposed in England to provide computer equipment and connectivity for families with DMD to reduce social isolation and to create a window into the world [28]. However, not all families were willing to participate in the program at that time. Since then, significant progress has been made in the use of electronic devices, equipment, social media, and applications. Organizations like the Parent Project of Muscular Dystrophy have developed their websites [29]. Technological barriers are diminishing, as mobile computing equipment becomes smaller and more affordable and wireless connectivity becomes more reliable and widespread. Currently, smartphones offer many possibilities to improve communication, access, and participation, and give a unique opportunity to directly deliver functionality to people with disabilities and their caregivers.

### Applications of Telemedicine in Different Areas of DMD Health Care

#### Neurology

Neurological examinations for DMD require personal contact between doctors and patients, making virtual examinations during teleconsultations challenging [30]. Available data comparing virtual examinations to traditional physical examinations are limited, and standardized protocols for televisits are lacking in the literature [31-34].

To address these challenges, researchers have developed their televisit systems and protocols. Structured surveys such as the Brook and Vignos scales or Egen Scale Classification used for clinical evaluation and functionality can be useful for collecting standardized data [35-38]. Additionally, other scales commonly used in clinical trials (the 6-Minute Walk Test, Quantitative Muscle Testing, the DMD Functional Ability Self-Assessment Tool, or the Performance of the Upper Limb Scale) can be utilized in telemedicine for assessing muscle function and progression of disease [39-41]. For children or adult patients with advanced-stage DMD, the presence of a telepresenter during the virtual examination can be helpful [17,28].

Although telemedicine became prevalent during the pandemic, hybrid programs combining telemedicine with in-person care emerged after the pandemic. Nowadays, it is recommended that the initial diagnostic visit for patients with DMD takes place in-person at the center, with possible follow-up teleconsultations [42,43].

#### Pulmonology

Respiratory function is crucial for the quality and length of life of patients with DMD; therefore, annual evaluation of respiratory function is recommended for patients with gait preservation and every 6 months for those without or even more frequent if clinical symptoms dictate [44]. This includes collecting information on the type, number, and duration of respiratory infections, presence of cough reflex, daytime hypoventilation symptoms and sleep quality, as well as pulmonary function tests, including spirometry, peak cough flow, respiratory muscle strength (sniff nasal inspiratory pressure, maximal static inspiratory and expiratory pressures), and sleep study (polysomnography) with overnight capnography. Some of these procedures can be supported by telemedicine, for example, during virtual visits, health care professionals can provide information on respiratory care and collect data on chest infections and symptoms of hypoventilation or ineffective sleep (preferably using validated questionnaires, eg, Sleep-Disordered Breathing Questionnaire In Neuromuscular Patients [SiNQ‑5]) [45].

In a case series involving 3 patients with neuromuscular disorders—including one with DMD—video consultations, supported by baseline physiological monitoring, facilitated early identification of respiratory complications and helped avoid hospitalization [46]. Further, telemonitoring of pulmonary function by using portable devices at home (e-spirometry with assessment of forced vital capacity), in addition to traditional spirometry in reference centers, can be employed in patients with DMD [47,48].

Web-based care services could potentially be beneficial for adolescent and young adults in the later stages of the disease who require ventilatory support, aiding in decision-making and providing timely interventions for respiratory issues. A study conducted during the pandemic found that weekly video consultations (averaged 23 minutes) and ventilator telemonitoring successfully addressed most problems related to general clinical status and ventilation in a group of 21 children (including 5 with neuromuscular diseases), who relied on long-term ventilatory support (noninvasive and invasive) and living at home [49]. That study also identified challenges with telemedicine, particularly the absence of sufficient technology at home, such as low internet signal or lack of internet connectivity, which affected 17% of the participants [49]. Studies presenting comprehensive virtual respiratory care dedicated exclusively to patients with DMD are generally lacking. However, data from pediatric patients with other conditions requiring long-term respiratory support at home indicate that although clinical complications are common and respiratory decompensation is the most common cause, telemedicine can facilitate early diagnosis and treatment of life-threatening events [50]. In addition, it has been shown to reduce the frequency of hospitalizations and improve caregivers’ sense of security and confidence in home care among ventilated patients [51].

#### Cardiology

DMD can lead to significant cardiac complications, including cardiomyopathy, arrhythmias, and heart failure, which are all potentially lethal [52,53]. Early diagnosis and treatment of cardiomyopathy leads to favorable ventricular remodeling [54]. Because adverse myocardial changes can occur at a younger age than previously thought and due to the importance of diagnosis before overt cardiac dysfunction, yearly cardiac screening is recommended [54]. Cardiac diagnostics with electrocardiography, echocardiography, Holter monitoring, and magnetic resonance imaging require in-person visits. However, telemedicine can provide access to specialized care for patients who may not have local access to cardiologists [55]. Telemedicine enables remote monitoring of cardiac function and arrhythmias by using wearable sensors and implantable devices, allowing for timely interventions to prevent or treat cardiac complications [56].

#### Nutrition

Proper nutrition is vital for patients with DMD. Eating disorders at the early stage of the disease due to steroid therapy and immobilization cause concern for excessive body weight. At later stages of the disease, there is a risk of malnutrition due to the weakening of the muscles of the gastrointestinal tract [4]. Teleconsultations can involve assessing the nutritional status, monitoring diet and fluid intake, and providing nutritional counseling. Web-based surveys such as the Food Frequency Questionnaire-6 item questionnaire [57] are useful in collecting dietary data. Software applications can assist patients in monitoring their dietary goals and measurements [58]. These interventions can address both excessive body weight in the early stages of the disease and the risk of malnutrition in later stages.

#### Psychology

Psychological support is needed at every stage of chronic disease. Studies [59] have shown that psychological and peer support via telehealth programs such as video sessions are useful and helpful for people with DMD and their caregivers. Telehealth programs can include health promotion causing improvement in the health-related quality of life. One study demonstrated the benefits of virtually delivered health coaching sessions (the first session was face-to-face, followed by 4 virtual sessions) related to physical activity and diet for males with DMD [60].

#### Telerehabilitation

According to the World Physiotherapy and the International Network of Physical Therapy Regulatory Authorities, the purpose of digital physical therapy practice is to facilitate the effective delivery of physical therapy services by improving access to care and information and managing health care resources [61]. Telerehabilitation consists of consulting, diagnostics, and therapy conducted with the use of interactive digital technologies in real-time connection (synchronous) or through prerecorded videos (asynchronous). Caregiver involvement is crucial for individuals with DMD to ensure the proper execution of exercises during telerehabilitation [62]. Web-based video instructions are more acceptable than live workshops [63]. Another study showed that 8-week telerehabilitation sessions enhanced muscle strength in ambulatory patients with DMD and that regular web-based meetings with physiotherapists were more effective than prerecorded or streaming video exercises [64]. Telerehabilitation can be supported by telemonitoring in patients with DMD (eg, cardiovascular parameters with electrocardiogram [65], overall physical activity level, step activity with accelerometers [66], effects of respiratory rehabilitation with home spirometers [47]). Virtual reality, including video games, has proven to be a valuable assistive technology for home-based rehabilitation [47].

#### Terminal Phase

Some studies [67-69] have shown that telemedicine can be used at every stage of chronic disease, including patients in the terminal phase of the disease, both in adults and children. This is important because patients with DMD can present hospice phase and have a premature death at a young age. Hospice programs utilizing telemedicine have mostly focused on children with oncological diseases, but the benefits extend to patients with neuromuscular diseases even in the form of simple teleconsultations [67-73]. Devices with touch screens, large letters, and easy-to-understand pictograms have proven useful in telemedicine for patients in the terminal phase [74]. Despite this, patients with end-stage DMD (requiring always multidimensional palliative support) remain underrepresented in telehealth research. Formative studies are needed to develop and evaluate integrated telemedicine approaches that address the specific needs of these patients.

Home health and nursing services may complement telemedicine in end-stage DMD by enabling in-home assessments, medication administration, and laboratory sample collection. This type of care can serve as an effective bridge between virtual supervision and hands-on clinical support, ensuring continuity for patients with complex needs. Evidence from other chronic conditions shows that nurse-led telehomecare can reduce hospitalizations and improve the quality of life [75,76]. However, this hybrid approach remains underexplored in neuromuscular disorders. Future studies should evaluate its feasibility by using indicators such as hospital admissions, clinical stability, and caregiver satisfaction.

### Summary

Telemedicine provides numerous benefits for individuals with DMD, their caregivers, and health care professionals. It facilitates seamless communication among medical professionals from different centers, resulting in faster information exchange and the sharing of valuable experiences. By creating communities on social media platforms, individuals with DMD and their caregivers can find mutual support and gain a broader perspective of the world. Telemedicine has found applications in many areas of DMD health care, including neurology, pulmonology, cardiology, dietetics, psychology, and even terminally ill patients. Telerehabilitation with virtual reality can be especially attractive to patients with DMD.

A limitation of telemedicine is the absence of standardized televisit protocols and a comprehensive virtual physical examination, including neurological assessments. As a result, the initial diagnostic visit necessitates in-person presence. Telerehabilitation is also not free of limitations. For example, it requires the caregiver’s assistance, and for optimal outcomes, real-life physiotherapy cannot be fully replaced by web-based treatment. Technical barriers and privacy concerns can also limit the use of telemedicine. Although there is no substitute for personal contact with a doctor, it seems that patients with DMD can benefit greatly from digital health. Furthermore, patients with DMD and their family caregivers are relatively young and therefore can more easily adapt to the daily use of technology.

## Clinical Consequences

Telemedicine cannot replace face-to-face visits—especially the first diagnostic visit must take place at the hospital. Telemedicine can support DMD health care when hospital visits are not possible. Telemedicine can be helpful as a window to the world for patients and their families and a quick way to exchange information and consultations between medical centers.

## Abbreviations

DMD: Duchenne muscular dystrophy
EURO-NMD: European Reference Network for the thematic grouping of rare Neuromuscular Diseases
SiNQ‑5: Sleep-Disordered Breathing Questionnaire In Neuromuscular Patients
TelePALS: Telemedicine for People with Amyotrophic Lateral Sclerosis
WHO: World Health Organization
